# Noninvasive multimodal imaging in diagnosing polypoidal choroidal vasculopathy

**DOI:** 10.1186/s12886-019-1244-5

**Published:** 2019-11-16

**Authors:** Jingyuan Yang, Mingzhen Yuan, Erqian Wang, Song Xia, Youxin Chen

**Affiliations:** 1 Department of Ophthalmology, Peking Union Medical College Hospital, Peking Union Medical College Hospital, Chinese Academy of Medical Sciences, No.1 Shuaifuyuan, Wangfujing, Dongcheng District, Beijing, 100730 China; 2 Key Laboratory of Ocular Fundus Diseases, Chinese Academy of Medical Sciences, Beijing, China; 3 Department of Ophthalmology, Guizhou Provincial People’s Hospital, Guiyang, Guizhou China

**Keywords:** Age-related macular degeneration, Diagnosis, Imaging, Polypoidal choroidal vasculopathy

## Abstract

**Purpose:**

To investigate the diagnostic accuracy of noninvasive multimodal imaging methods in diagnosing polypoidal choroidal vasculopathy (PCV) and distinguishing PCV from typical neovascular age-related macular degeneration (nvAMD).

**Methods:**

Retrospective study. Imaging features of noninvasive multimodal imaging methods, including fundus photography (FP), B-scan optical coherence tomography (OCT), en face OCT, OCT angiography, and autofluorescence, of 103 eyes with PCV or typical nvAMD were reviewed. Diagnostic strategy was established based on imaging features and was validated in other 105 eyes with PCV or typical nvAMD.

**Results:**

Features of subretinal orange nodule on FP, thumb-like PED on OCT, notched PED on OCT, bubble sign on OCT, and Bruch’s membrane depression under serosanguinous PED on OCT were more common. When the diagnostic strategy of using at least 2 of 5 features was performed, there is 0.88 sensitivity and 0.92 specificity for diagnosing PCV. The results of the validation test further confirmed the diagnostic strategy with 0.94 sensitivity and 0.93 specificity.

**Conclusions:**

Noninvasive multimodal imaging, especially FP and B-scan OCT, provide high sensitivity and specificity for diagnosing PCV and distinguishing PCV from typical nvAMD, when at least 2 of 5 suggestive imaging features are present.

## Background

Polypoidal choroidal vasculopathy (PCV) is an important cause of visual loss in elderly, which is characterized with the presence of polypoidal or aneurysmal hyperfluorescent with or with branching vascular network (BVN) on indocyanine green angiography (ICGA) [1]. Although it is regarded as a subtype of neovascular age-related macular degeneration (nvAMD), PCV has differences on the natural history, treatment regimens, and prognosis with typical nvAMD [1–6]. Therefore, to differentiate PCV from typical nvAMD can determine the recommendations for patients regarding management and prognosis in clinical practice. Although ICGA is the gold standard to diagnose PCV [1, 7], it is an invasive imaging method, and it is contraindicated in patients with a history of allergy to iodine-based dye [8]. What’s more, in real world ICGA is not always available to perform, especially in many areas in the developing countries. Therefore, to diagnose PCV using noninvasive imaging methods might help in clinical practice. Multimodal imaging criteria has been regarded as the aim and direction of future PCV diagnostic criteria [1].

Previous studies have reported the high sensitivity and specificity of noninvasive imaging methods in detecting PCV, including optical coherence tomography [9–11]. However, ophthalmologists are pretend to get information from complicated examinations of fundus rather than optical coherence tomography angiography (OCT) alone in clinical practice. Chaikitmongkol et al. reported multimodal imaging methods to diagnose PCV, in which an invasive imaging method of fluorescent angiography (FA) was used [12, 13]. In clinical practice, ophthalmologists can obtain clues suggesting the diagnosis of PCV using noninvasive imaging methods, including the presence of subretinal orange nodules on fundus photography (FP) and the presence of hyper-reflectivity ring on OCT B-scans and en face OCT. Therefore, a provisional diagnosis of PCV might be made based on noninvasive imaging methods without ICGA.

This study aims to investigate sensitivity, specificity, and predictive accuracy of noninvasive multimodal imaging methods, including FP, B-scan OCT, en face OCT, optical coherence tomography angiography (OCTA), and autofluorescence (AF), to diagnose PCV without using ICGA and to differentiate PCV from typical nvAMD.

## Methods

### Data collection

This study was approved by the Institutional Review Boards of Peking Union Medical College Hospital (reference number S-K631). All patients provided written informed consent when being performed the examinations of FA and ICGA. Patients who presented to Peking Union Medical College Hospital between January 1, 2016 and February 28, 2019, with newly diagnosed PCV and typical nvAMD in unilateral or bilateral eyes. The diagnosis of PCV or typical nvAMD based on these imaging methods was made according to current guidelines by 2 expert retinal specialists (YC and SX) who were masked to the results of other examinations [1]. Disagreements were resolved by open adjudication between the 2 authors. One hundred and three eyes of 82 patients were enrolled. The diagnosis of PCV was made based on hyperfluorescent polypoidal or aneurysmal lesions with or without branching vascular network on ICGA. The diagnosis of typical nvAMD was made based on neovascular lesions on FA without polypoidal or aneurysmal lesions on ICGA. These patients also underwent 5 noninvasive imaging examinations, including FP, B-scan OCT, en face OCT, OCTA, AF within 3 days after the examinations of FA and ICGA. OCT included at least 25 cross-sectional B-scan images. Exclusion criteria included: 1) a history of other ocular diseases, including secondary choroidal neovascular diseases, diabetic retinopathy, pathological myopia, uveitis, etc.; 2) poor image quality or loss of imaging details of lesions because of cloudy refracting media and unstable fixation; 3) any systematic disorders that affect the eyes.

### Evaluation of noninvasive imaging features

The diagnostic imaging features of noninvasive multimodal imaging, including FP, OCT, en face OCT, OCTA, and AF, were evaluated by 2 independent retinal specialist graders (JY and MY) who were masked to the results of FA and ICGA. In cases with disagreement, a third retinal specialist (EW) made the final decision. Noninvasive diagnostic strategy for PCV was established based on abovementioned noninvasive multimodal imaging. The diagnostic noninvasive imaging features were defined according to the published literature. Diagnostic features of clinical clues for PCV on FP included subretinal orange nodules, hemorrhagic pigment epithelial detachment (PED), multiple lesions, extensive hemorrhage with area more than 4 disc areas, and absence of drusen [7]; on OCT, multiple PED, thumb-like PED, notched PED, double-layer sign, pachychoroid, and depression of Bruch’s membrane under serosanguinous PED [14–16]; on en face OCT, dilated choroidal vessel, hyper-reflective ring adjacent to and beneath retinal pigment epithelium (RPE), hyper-reflective foci, and RPE ring [17, 18]; on OCTA, abnormal vessel under RPE on en face OCTA, and abnormal blood flow resembling polyp on both en face OCTA and cross-sectional OCTA [19, 20]; on AF, hyperfluorescent ring, and granular hypofluorescence (Additional file 1: Figure S1) [21, 22]. No missing data existed.

### Evaluation of diagnostic ability of noninvasive imaging features

The diagnostic ability of each noninvasive imaging feature was evaluated, and the noninvasive imaging features with high diagnostic ability were selected as major diagnostic criteria. Various combinations of different amounts of major diagnostic criteria were included in diagnostic test. The combination of a certain amount of major diagnostic criteria which has the best diagnostic ability was used as the noninvasive diagnostic strategy for PCV.

To validate the efficacy of the diagnostic strategy, the medical records of another 105 eyes from 85 patients with PCV or typical nvAMD who presented from January 1, 2014 to December 31, 2015 were retrospectively reviewed. All the enrolled eyes for validation have been performed FA, ICGA, and the examinations which were used in the diagnostic strategy. The diagnosis of PCV or typical nvAMD was also made by 2 expert retinal specialists (YC and SX) who were masked to the results of other examinations. Two independent retinal specialist graders (JY and MY) who were masked to the results of FA and ICGA used the diagnostic strategy on the enrolled eyes to diagnose PCV, and a third retinal specialist (EW) also made the final decision in cases with disagreement. The diagnostic ability of the diagnostic strategy was validated.

### Statistical analysis

To determine the intended sample size, the values of sensitivity (0.95) and prevalence (0.5) was set according to the most recent studies of Chinese population and previous diagnostic study on PCV [1, 13, 23, 24]. And the precision of estimate was set as 0.1. Therefore, the sample size should be no less than 36.5 [25]. Both diagnostic test and validation test meet the requirement of sample size.

Parameters of diagnostic ability, including sensitivity, specificity, and predictive accuracy using the area under the receiver operating characteristic curve (AUC) of each feature on FP, OCT, en face OCT, OCTA, and AF to diagnose PCV were determined. Given that the perfect score of predictive accuracy of AUC is 1, and the evaluated potential diagnostic features with an AUC of 0.8 or more were regarded as major criteria with high accuracy for diagnosis of PCV using noninvasive imaging methods. The features regarded as major criteria were calculated to determine the optimal number of features to diagnose PCV.

## Results

This study included 103 eyes of 82 Chinese patients, and the mean (standard deviation) age was 65.13 (7.50) years. Of the 103 eyes, the definitive diagnosis by the expert specialists was PCV in 52 eyes, and typical nvAMD in 51 eyes.

The sensitivity, specificity, and AUC for the potential diagnostic features detected in noninvasive multimodal imaging methods are shown in Table 1, and detailed information was summarized in Additional file 2: Table S2. On FP, the diagnostic feature of a subretinal orange nodule had high accuracy. Several diagnostic features of thumb-like PED, notched PED, bubble sign, and Bruch’s membrane depression under serosanguinous PED on OCT also had high accuracy. No features detected using en face OCT, OCTA, and AF showed an AUC of 0.8 or higher.

**Table 1:** Sensitivity, specificity, and predictive accuracy of prespecified potential diagnostic features detected using noninvasive multimodal imaging methods

Feature	Sensitivity (95% CI)	Specificity (95% CI)	AUC (95% CI)
Fundus photograph			
Subretinal orange nodule	0.78 (0.65–0.88)	0.96 (0.85–0.99)	0.88 (0.80–0.95)
Hemorrhagic PED	0.54 (0.40–0.68)	0.98 (0.88–1.00)	0.76 (0.66–0.86)
Multifocal lesions	0.12 (0.05–0.24)	1.00 (0.91–1.00)	0.56 (0.45–0.67)
Extensive hemorrhage	0.27 (0.16–0.41)	1.00 (0.91–1.00)	0.64 (0.53–0.74)
Absence of drusen	0.67 (0.53–0.79)	0.84 (0.71–0.93)	0.76 (0.66–0.85)
Optical coherence tomography			
Multiple PED	0.81 (0.67–0.90)	0.64 (0.50–0.77)	0.73 (0.63–0.83)
Thumb-like PED	0.73 (0.59–0.84)	0.90 (0.78–0.96)	0.82 (0.73–0.90)
Notched PED	0.75 (0.61–0.86)	0.88 (0.75–0.95)	0.82 (0.73–0.90)
Double-layer sign	0.83 (0.69–0.91)	0.55 (0.40–0.69)	0.69 (0.58–0.79)
Bubble sign	0.73 (0.59–0.84)	0.94 (0.83–0.98)	0.84 (0.75–0.92)
Pachychoroid	0.63 (0.49–0.76)	0.92 (0.80–0.97)	0.78 (0.69–0.87)
Bruch’s membrane depression	0.77 (0.63–0.87)	0.92 (0.80–0.97)	0.85 (0.77–0.93)
En face optical coherence tomography			
Dilated choroidal vessel	0.37 (0.24–0.51)	0.88 (0.75–0.95)	0.62 (0.52–0.73)
Hyper-reflective ring adjacent to and beneath RPE	0.67 (0.53–0.79)	0.78 (0.64–0.88)	0.73 (0.63–0.83)
Hyper-reflective foci	0.40 (0.27–0.55)	0.73 (0.58–0.84)	0.57 (0.45–0.68)
RPE ring	0.90 (0.78–0.96)	0.47 (0.33–0.61)	0.69 (0.58–0.79)
Optical coherence tomography angiography			
Abnormal vessel under RPE	0.96 (0.86–0.99)	0.10 (0.04–0.22)	0.53 (0.42–0.64)
Abnormal blood flow resembling polyp	0.52 (0.38–0.66)	0.86 (0.73–0.94)	0.69 (0.59–0.79)
Autofluorescence			
Hyperfluorescent ring	0.42 (0.29–0.57)	0.92 (0.80–0.97)	0.67 (0.57–0.78)
Granular hypofluorescence	1.00 (0.91–1.00)	0 (0–0.09)	0.50 (0.39–0.61)

*AUC* area under curve, *CI* confidence interval, *PED* pigment epithelial detachment, *RPE* retinal pigment epithelium

**Fig. 1:**
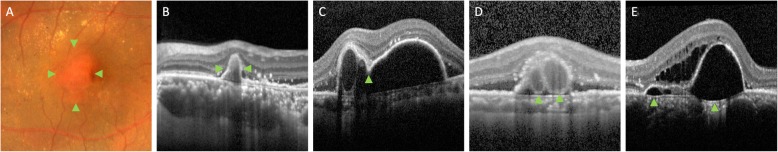
Typical potential diagnostic features detected using fundus photography (FP) and B-scan optical coherence tomography (OCT) that suggest the diagnosis of polypoidal choroidal vasculopathy. These 5 features (green arrowhead), including subretinal orange nodule on FP (**a**), thumb-like pigment epithelial detachment (PED) on OCT (**b**), notched PED on OCT (**c**), bubble sign on OCT (**d**), and Bruch’s membrane depression under serosanguinous PED on OCT (**e**), were used as major criteria in the study

The sensitivity and specificity of various combinations of these 5 potential diagnostic features (Fig. 1) with AUC of 0.8 or higher were used for diagnosis of PCV can be seen in Table 2. When at least 2 of 5 major criteria were used for diagnosing PCV, the predictive accuracy (AUC) has the highest value of 0.90, with 0.88 sensitivity, 0.92 specificity, 0.92 positive predictive value, and 0.89 negative predictive value.

**Table 2:** Sensitivity, specificity, positive predictive value, negative predictive value, and diagnostic accuracy of multiple major criteria

Major criteria	Sensitivity (95% CI)	Specificity (95% CI)	PPV (95% CI)	NPV (95% CI)	AUC (95% CI)
≥ 1 of 5 major criteria	0.92 (0.81–0.98)	0.75 (0.60–0.85)	0.79 (0.66–0.88)	0.90 (0.76–0.97)	0.83 (0.75–0.92)
≥ 2 of 5 major criteria	0.88 (0.76–0.95)	0.92 (0.80–0.97)	0.92 (0.80–0.97)	0.89 (0.76–0.95)	0.90 (0.84–0.97)
≥ 3 of 5 major criteria	0.81 (0.67–0.90)	0.96 (0.85–0.99)	0.95 (0.83–0.99)	0.83 (0.71–0.91)	0.88 (0.81–0.96)
≥ 4 of 5 major criteria	0.69 (0.55–0.81)	0.98 (0.88–1.00)	0.97 (0.84–1.00)	0.76 (0.63–0.85)	0.84 (0.75–0.92)
5 of 5 major criteria	0.46 (0.32–0.60)	1.00 (0.91–1.00)	1.00 (0.83–1.00)	0.65 (0.53–0.75)	0.73 (0.63–0.83)

*AUC* area under curve, *CI* confidence interval, *NPV* negative predictive value, *PPV* positive predictive value

**Fig. 2:**
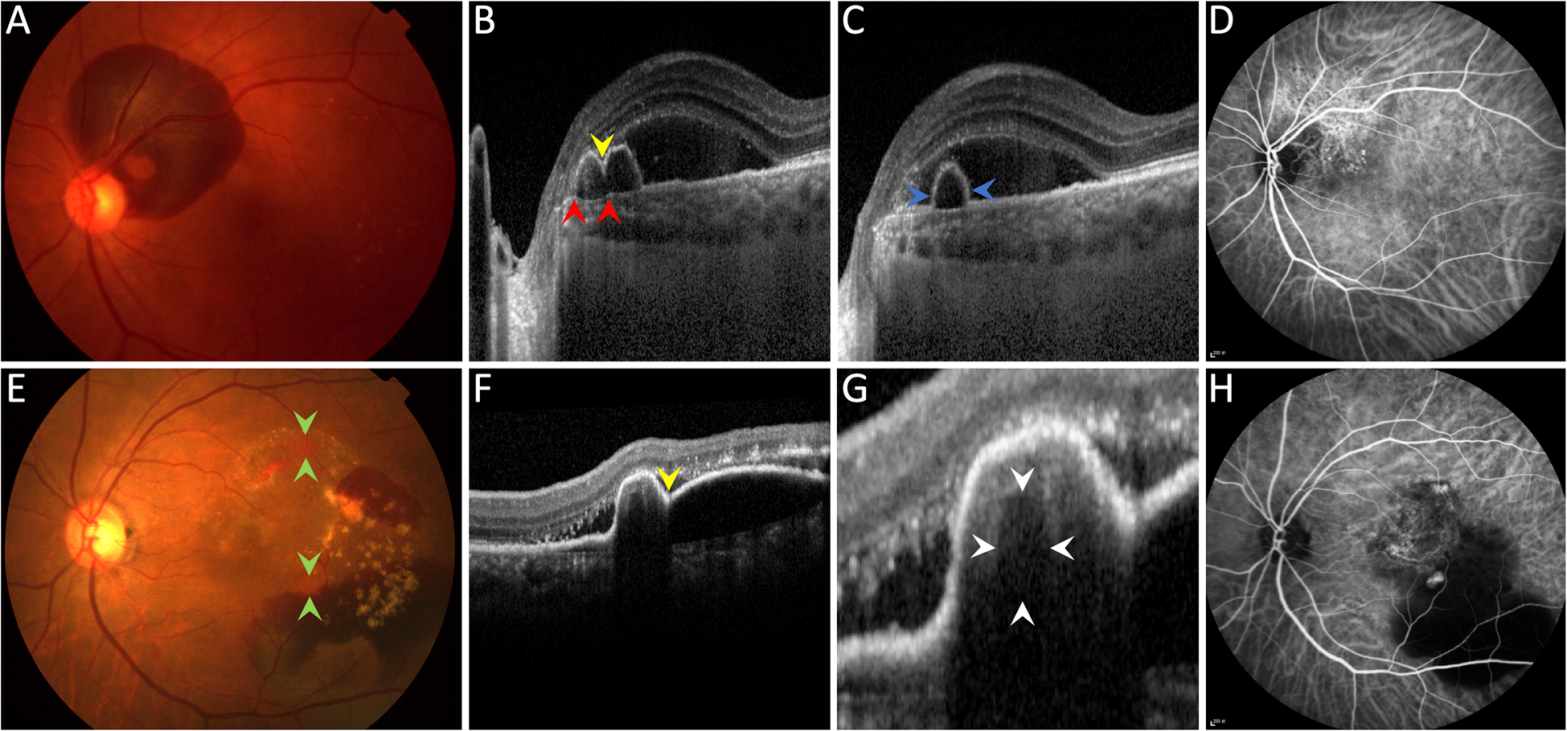
Examples of noninvasive multimodal imaging in diagnosing polypoidal choroidal vasculopathy (PCV). Top row: An elderly man had peripapillary hemorrhage in his left eye (**a**). Optical coherence tomography (OCT) shows notched PED (yellow arrowhead in **b**), Bruch’s membrane under serosanguinous PED (between red arrowheads in **b**), and thumb-like PED (between blue arrowheads in **c**). Indocyanine green angiography (ICGA) confirms the diagnosis of PCV (**d**). Bottom row: An elderly woman had orange nodules (between green arrowheads in **e**), notched PED (yellow arrowhead in **f**), and suspected bubble sign (between white arrowheads in **g**). ICGA confirms the diagnosis of PCV (**h**)

A total of 105 eyes, including 50 eyes with PCV and 55 eyes with typical nvAMD, were retrospectively enrolled for validating the diagnostic strategies of using various combinations of major criteria. The diagnostic strategy of using at least 2 of 5 major criteria still has the highest predictive accuracy (AUC) of 0.93 (95% CI 0.88–0.99), with 0.94 sensitivity (95% CI 0.82–0.98) and 0.93 specificity (95% CI 0.82–0.98) (Fig. 2).

## Discussion

The present study finds diagnostic strategy for PCV using noninvasive multimodal imaging method rather than invasive ICGA. This study suggested that when at least 2 of 5 highly potential diagnostic features detected using FP and OCT were present, there was 0.88 sensitivity, 0.92 specificity, and 0.90 AUC of predictive accuracy for diagnosing PCV. The results of the validation test further confirmed the diagnostic criteria with 0.94 sensitivity and 0.93 specificity. The 5 highly potential diagnostic features included subretinal orange nodule on FP, thumb-like PED on OCT, notched PED on OCT, bubble sign on OCT, and Bruch’s membrane depression under serosanguinous PED on OCT, which was firstly noticed in the current study. These features suggested a diagnosis of PCV rather than typical nvAMD, which should be looked for when PCV was supposed to be differentiate from typical nvAMD. One benefit of considering these 5 major criteria to identify PCV without ICGA is that noninvasive multimodal imaging could be accessed quicily and easily in most clinics, and FP and OCT could be routinely performed in most areas in the world.

Differentiating PCV from typical nvAMD is desirable since the diagnosis of PCV is essential for patients regarding management and prognosis. PCV was regarded as a subtype of nvAMD characterized by aneurysmal type 1 choroidal neovascularization. Recently the feature of pachychoroid in PCV eyes led some investigators to recommend PCV falls within the pachychoroid spectrum, which might have a different cause of AMD [26]. As the pathogenesis and clinical features of PCV are distinct from those of nvAMD, the management of PCV consist a wide spectrum of treatment options, including verteporfin photodynamic therapy (PDT), anti-vascular endothelial growth factor (VEGF) therapy, focal laser photocoagulation, and various combinations of these therapies. On the contrary, intravitreal anti-VEGF therapy was the mainstay of treatment for typical nvAMD. When compared with typical nvAMD, the prognosis of PCV varies with various clinical and imaging features and the choice of treatment regimens in clinical practice [1, 26, 27]. Therefore, it is crucial to distinguish PCV from typical nvAMD accurately.

Diagnosis of PCV using noninvasive imaging methods is needed in clinical practice. Although there is no universally accepted definition of PCV currently, the presence of subretinal focal hyperfluorescence on ICGA was believed to be essential to diagnose PCV [1, 28]. However, the clinical application of ICGA is limited because of its invasiveness and possibility of allergy [8]. Besides, PCV may be underdiagnosed in people whose ancestry is not Asian or African because ICGA might not be routinely performed. What’s more, ICGA is not accessible or available in some areas, especially in less developed areas where a majority of individuals are Asian and African. It might be helpful if noninvasive diagnostic criteria were supplemented to the invasively diagnostic criteria of PCV using ICGA. Therefore, previous studies have investigated noninvasive diagnostic criteria for PCV using OCT alone. De Salvo et al. reviewed 51 eyes with PED attributable to either PCV or occult choroidal neovascularization, and reported 0.946 sensitivity and 0.929 specificity of diagnosing PCV using spectral-domain OCT. [9] Liu et al. reported 0.875 sensitivity and 0.862 specificity on distinguishing PCV from typical nvAMD using OCT in a prospective study, and the OCT features, included PED, double-layer sign, and thumb-like PED, suggested a diagnosis of PCV [10]. However, these studies did not consider the potential prognostic information provided by other noninvasive imaging methods. Although additional en face OCT, OCTA, and AF information did not help improve the predictive accuracy, they still provide several imaging features of PCV, which might suggest potential PCV diagnosis.

Noninvasive multimodal imaging methods were used in the present study. On the contrary, Chaikitmongkol et al. reported a hybrid diagnostic strategy of noninvasive FP and OCT, and invasive FA. They reported diagnostic criteria with 0.95 sensitivity and 0.95 specificity, which used at least 2 of 4 signs, included hemorrhagic PED on FP, notched PED on OCT, sharply peaked PED (also known as thumb-like PED in the current study) on OCT, and hyperreflective ring (also known as bubble sign in the current study) on OCT [12, 13]. They did not consider potential diagnostic value of other imaging methods as well, although they also chose FP and OCT in their diagnostic criteria, which was same as our diagnostic strategy. Compared with the current study, the diagnostic feature of subretinal orange nodules in their study had a low sensitivity of 0.39, which was totally different from the sensitivity value of 0.78 in the current study. The orange-red elevated lesions on FP was regarded a major diagnostic criterion by the proposed guidelines by the Japanese Study Group of Polypoidal Choroidal Vasculopathy [29]. PCV has different clinical features in various ethnic groups [30]. One of possible explanations is that Chinese PCV patients might share more common clinical presentations with Japanese patients than patients in Thailand. Besides, because the color of subretinal orange nodules is similar to the orange-reddish appearance of fundus with pachychoroid diseases [31], experienced graders were necessary to determine the occurrence of subretinal orange nodules in clinical practice.

In the present study, we came up with a new sign of PCV, the Bruch’s membrane depression under serosanguinous PED. However, the pathogenesis of this sign is unknown. The line of Bruch’s membrane on OCT was more likely to be curved and protruded to the direction of choroid in eyes with PCV, which suggested that the hydrostatic pressure in serosanguinous PED might be higher in PCV than that in typical nvAMD, and the abnormal vessels in PCV lesions might have higher pressure than peripherally normal choroidal vessels. Besides, the increased hydrostatic pressure might also correlate with dilated choroidal vessels and breakthrough vitreal hemorrhage in eyes with PCV [32, 33]. Although an OCT feature of PCV was newly noticed, the pathogenesis of Bruch’s membrane depression needs further investigation.

In the current study, the sensitivity and specificity were slightly higher in the validation test than the values in the diagnostic test, which might be owing to different sample selections. However, all the values were satisfying, which give us confidence that these diagnostic criteria will probably meet the requirements for clinical work.

We did not intend to attenuate the significance of ICGA, since ICGA is still the gold standard on diagnosis of PCV [1, 7]. However, it is well known that future diagnostic criteria should aim to include multimodal imaging criteria [1]. Besides, when PDT with verteporfin is considered in combination with anti-VEGF agents, ICGA still may be needed to identify lesions to guide PDT, although further studies are needed to investigate whether various anti-VEGF agents combined with PDT provides superior visual acuity results with fewer injection numbers compared with anti-VEGF monotherapy [1].

The limitations of this study include its retrospective design in a single center with limited sample size. Besides, only Chinese patients were enrolled. The diagnostic criteria remain to be validated in other Asian or African populations, for whom PCV is endemic, or even in Caucasian population with PCV. Moreover, the recognition of the imaging features described in the current study requires expertise in PCV and imaging interpretation. Additionally, only treatment-naïve eyes were enrolled in this study, and it remains uncertain whether the diagnostic criteria could be generalized on eyes that have undergone previous treatment.

In conclusion, this study suggests that potential diagnostic features detected using noninvasive multimodal imaging methods, especially using FP and OCT, on Chinese individuals with typical nvAMD and PCV provide high sensitivity and specificity to diagnose PCV. The presence of at least 2 of 5 signs of subretinal orange nodule on FP, thumb-like PED on OCT, notched PED on OCT, bubble sign on OCT, and Bruch’s membrane depression under serosanguinous PED on OCT was used to diagnose PCV and to differentiate PCV from typical nvAMD. Further studies of multimodal imaging criteria for diagnosing PCV on treated eyes with PCV and more ethnic groups are needed.

## Supplementary information

**Additional file 1:** **Figure S1**. Examples of noninvasive multimodal imaging features of polypoidal choroidal vasculopathy (PCV). **A:** Subretinal orange nodule (green arrowheads) on fundus photograph (FP). **B:** Extensive hemorrhagic pigment epithelial detachment (PED) on FP. **C:** Multifocal lesions (green arrowheads) without presence of drusen on FP. **D:** Multiple thumb-like PEDs on optical coherence tomography (OCT). **E:** Notched PED (green arrowhead) on OCT. **F:** Double-layer sign (between green arrowheads) on OCT. **G:** Bubble sign (between green arrowheads) on OCT. **H:** Pachychoroid (above green arrowheads) on OCT. **I:** Bruch’s membrane depression under serosanguinous PED (between green arrowheads) on OCT. **J:** Dilated choroidal vessel (green arrowhead) on en face OCT which was centered on the foveola. **K:** Multiple hyper-reflective ring adjacent to and beneath retinal pigment epithelium (RPE) (green arrowheads) on en face OCT. **L:** Multiple hyper-reflective foci (green arrowheads) on en face OCT. **M:** RPE ring (green arrowhead) on en face OCT. **N:** Abnormal vascular signal under RPE (green arrowhead) on OCT angiography (OCTA). **O:** Abnormal blood flow signal resembling polyps (green arrowheads) on OCTA. **P:** Hyperfluorescent ring (green arrowhead) on autofluorescence (AF). **Q:** Granular hypofluorescence (green arrowheads) on AF.

**Additional file 2: Table S2.** Presence of prespecified potential diagnostic features in PCV and nvAMD.

## Data Availability

The datasets used and/or analyzed during the current study are available from the corresponding author on reasonable request.

## Abbreviations

AF: Autofluorescence
AUC: Area under the receiver operating characteristic curve
FA: Fluorescent angiography
FP: Fundus photography
ICGA: Indocyanine green angiography
nvAMD: neovascular age-related macular degeneration
OCT: Optical coherence tomography angiography
OCTA: Optical coherence tomography angiography
PCV: Polypoidal choroidal vasculopathy (PCV)
PDT: Photodynamic therapy
PED: Pigment epithelial detachment
VEGF: Vascular endothelial growth factor
