# Supplementary figures and images for: Noninvasive multimodal imaging in diagnosing polypoidal choroidal vasculopathy
Source: BMC Ophthalmol. 2019 Nov 16;19:229. doi: 10.1186/s12886-019-1244-5 (PMC6858976; doi:10.1186/s12886-019-1244-5)

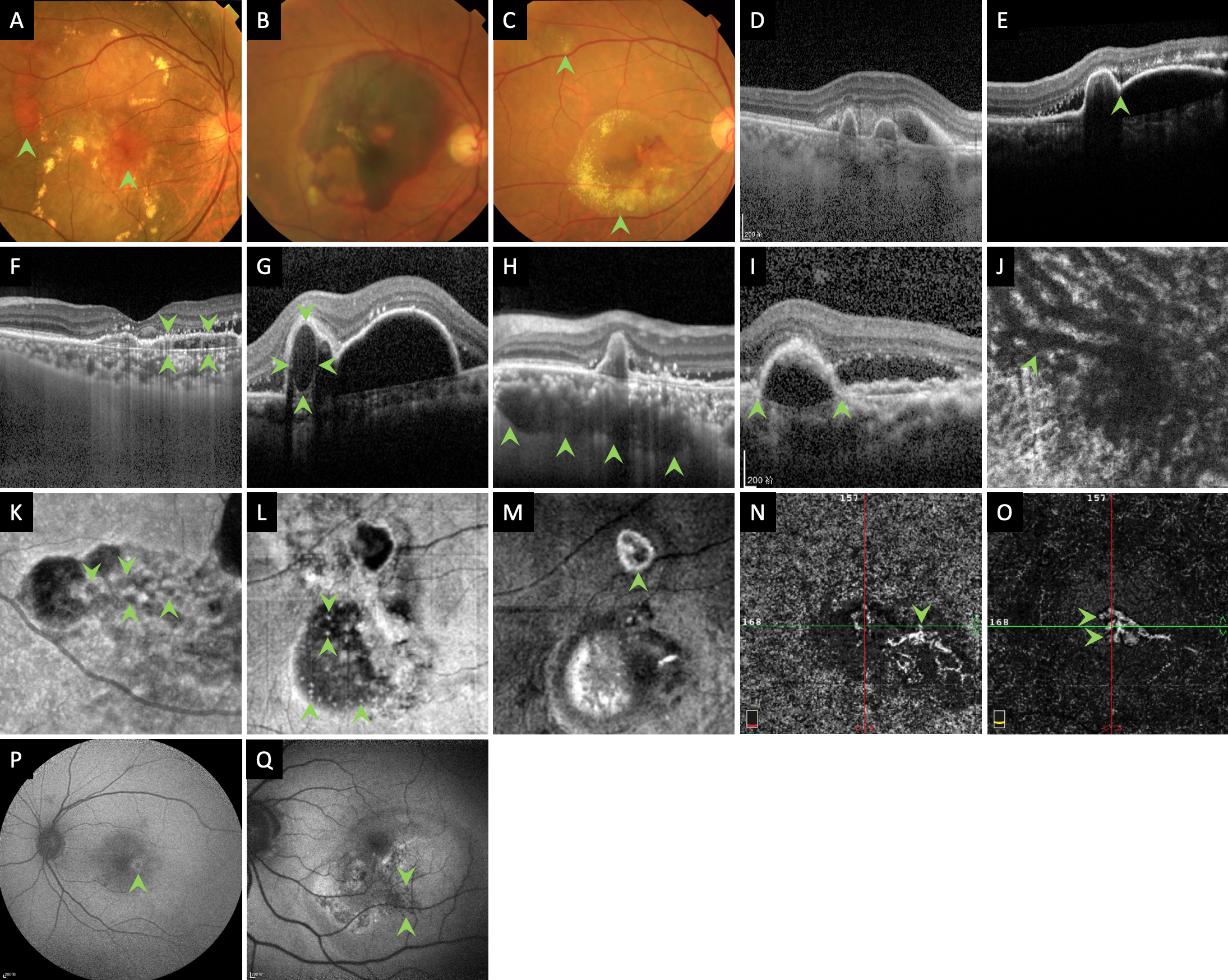

Supplement: Supplementary file 1 — Additional file 1: Figure S1. Examples of noninvasive multimodal imaging features of polypoidal choroidal vasculopathy (PCV). A: Subretinal orange nodule (green arrowheads) on fundus photograph (FP). B: Extensive hemorrhagic pigment epithelial detachment (PED) on FP. C: Multifocal lesions (green arrowheads) without presence of drusen on FP. D: Multiple thumb-like PEDs on optical coherence tomography (OCT). E: Notched PED (green arrowhead) on OCT. F: Double-layer sign (between green arrowheads) on OCT. G: Bubble sign (between green arrowheads) on OCT. H: Pachychoroid (above green arrowheads) on OCT. I: Bruch’s membrane depression under serosanguinous PED (between green arrowheads) on OCT. J: Dilated choroidal vessel (green arrowhead) on en face OCT which was centered on the foveola. K: Multiple hyper-reflective ring adjacent to and beneath retinal pigment epithelium (RPE) (green arrowheads) on en face OCT. L: Multiple hyper-reflective foci (green arrowheads) on en face OCT. M: RPE ring (green arrowhead) on en face OCT. N: Abnormal vascular signal under RPE (green arrowhead) on OCT angiography (OCTA). O: Abnormal blood flow signal resembling polyps (green arrowheads) on OCTA. P: Hyperfluorescent ring (green arrowhead) on autofluorescence (AF). Q: Granular hypofluorescence (green arrowheads) on AF. [file 12886_2019_1244_MOESM1_ESM.tif]
