# Supplementary material for: Noninvasive multimodal imaging in diagnosing polypoidal choroidal vasculopathy
Source: BMC Ophthalmol. 2019 Nov 16;19:229. doi: 10.1186/s12886-019-1244-5 (PMC6858976; doi:10.1186/s12886-019-1244-5)
Supplement: Supplementary file 2 — Additional file 2: Table S2. Presence of prespecified potential diagnostic features in PCV and nvAMD. [file 12886_2019_1244_MOESM2_ESM.docx]

**Additional file 2: Table S2** Presence of prespecified potential diagnostic features in PCV and nvAMD

| Feature | Presence in PCV | Absence in PCV | Presence in nvAMD | Absence in nvAMD |
| --- | --- | --- | --- | --- |
| Fundus photograph |  |  |  |  |
| Subretinal orange nodule | 41 | 11 | 2 | 49 |
| Hemorrhagic PED | 28 | 24 | 1 | 50 |
| Multifocal lesions | 6 | 46 | 0 | 51 |
| Extensive hemorrhage | 14 | 38 | 0 | 51 |
| Absence of drusen | 35 | 17 | 8 | 43 |
| Optical coherence tomography |  |  |  |  |
| Multiple PED | 42 | 10 | 18 | 33 |
| Thumb-like PED | 38 | 14 | 5 | 46 |
| Notched PED | 39 | 13 | 6 | 45 |
| Double-layer sign | 43 | 9 | 23 | 28 |
| Bubble sign | 38 | 14 | 3 | 48 |
| Pachychoroid | 33 | 19 | 4 | 47 |
| Bruch’s membrane depression | 40 | 12 | 4 | 47 |
| En face optical coherence tomography |  |  |  |  |
| Dilated choroidal vessel | 19 | 33 | 6 | 45 |
| Hyper-reflective ring adjacent to and beneath RPE | 35 | 17 | 11 | 40 |
| Hyper-reflective foci | 21 | 31 | 14 | 37 |
| RPE ring | 47 | 5 | 27 | 24 |
| Optical coherence tomography angiography |  |  |  |  |
| Abnormal vessel under RPE | 50 | 2 | 46 | 5 |
| Abnormal blood flow resembling polyp | 27 | 25 | 7 | 44 |
| Autofluorescence |  |  |  |  |
| Hyperfluorescent ring | 22 | 30 | 4 | 47 |
| Granular hypofluorescence | 17 | 35 | 17 | 34 |

nvAMD: neovascular age-related macular degeneration; PCV: polypoidal choroidal vasculopathy; PED: pigment epithelial detachment; RPE: retinal pigment epithelium.
